# Challenges and opportunities for ovarian cancer management in the epidemic of Covid-19: lessons learned from Wuhan, China

**DOI:** 10.1186/s13048-021-00784-2

**Published:** 2021-02-18

**Authors:** Zhilan Chen, Chun Zhang, Jiu Yin, Xin Xin, Hemei Li, Yapei Wang, Benjamin K. Tsang, Qinghua Zhang

**Affiliations:** 1 Department of Obstetrics and Gynecology, The Central Hospital of Wuhan, Tongji Medical College, Huazhong University of Science and Technology, No. 26 Shengli Street, Jiangan District, Wuhan, 430014 China; 2 Department of Obstetrics and Gynecology and Cellular and Molecular Medicine, University of Ottawa, and Chronic Disease Program, Ottawa Hospital Research Institute, Ottawa, Ontario Canada

**Keywords:** Covid-19, Ovarian cancer, Pandemic, Management

## Abstract

China and the rest of the world are experiencing an outbreak of the 2019 novel coronavirus disease (COVID-19). Patients with cancer are more susceptible to viral infection and are more likely to develop severe complications, as compared to healthy individuals. The growing spread of COVID-19 presents challenges for the clinical care of patients with gynecological malignancies. Ovarian debulking surgery combined with the frequent need for chemotherapy is most likely why ovarian cancer was rated as the gynecologic cancer most affected by COVID-19. Therefore, ovarian cancer presents a particular challenging task. Concerning the ovarian cancer studies with confirmed COVID-19 reported from large-scale general hospitals in Wuhan, we hold that the treatment plan was adjusted appropriately and an individualized remedy was implemented. The recommendations discussed here were developed mainly based on the experience from Wuhan. We advise that the management strategy for ovarian cancer patients should be adjusted in the light of the local epidemic situation and formulated according to the pathological type, tumor stage and the current treatment phase. Online medical service is an effective and convenient communication platform during the pandemic.

## Introduction

Severe acute respiratory syndrome coronavirus 2 (SARS-CoV-2), a novel coronavirus, emerged in the city of Wuhan, Hubei, China in early December, 2019 [48]. Since its first identification, coronavirus disease 2019 (COVID-19) has spread to over 200 countries with 103,402,619 cases and a total of 2,232,194 deaths worldwide (as of Februrary 01 52, 2021), according to the World Health Organization reports (https://www.who.int/emergencies/diseases/novelcoronavirus-2019/situation-reports). Since then, local and national governments have taken unprecedented measures in response to the COVID-19 outbreak. Wuhan realized zero growth of suspected or confirmed new cases on March 18, 2000. Subsequently, Wuhan was classified as a “low-risk” area on April 18. From the “storm center” of the epidemic to the “safest city in China” in the eyes of many academicians and experts, Wuhan has realized the fundamental reversal of the situation by implementing the national treatment strategies of “all those in need are tested, quarantined, hospitalized or treated”. Table 1 illustrates the chronology of major events of historical significance in relation to the pandemic.

**Table 1:** Chronology of major significant events during the pandemic in Wuhan

Date (2020)	Major events
January 23 rd (10:00 h)	City bus, subway, ferry, long-distance passenger transport, airport and railway station in Wuhan were temporarily closed
February 5th	Mobile cabin hospitals began to receive COVID-19 patients with mild symptoms.
February 4–8	Huoshenshan hospital and Leishenshan hospital were put into use to receive severe patients
February 18th	Cumulative number of confirmed cases reached the peak figure of 38,020
February 29th	The quarantine places, mobile cabin hospitals, and designated hospitals in Wuhan all had available extra beds for the first time
March 1st	The first closed mobile cabin hospital appeared in Wuhan
March 10th	All sixteen mobile cabin hospitals in Wuhan were closed.
March 13th	Zero increase of suspected case in Wuhan for the first time
March 18th	Zero increase of suspected case or confirmed case in Wuhan for the first time
March 25th	Wuhan was classified as “medium risk” area
April 6th	Zero increase of motality case
April 8th (0:00 h)	Wuhan city lifted the control measures. Wuhan had been closed for 76 days since 10:00,January 23.
April 18th	Wuhan was classified as “low risk” area as a whole
April 23rd	Severe COVID-19 cases in Wuhan were eliminated.
April 24th	The in-hospital COVID-19 patients in Wuhan were cleared.

Patients with cancer are more susceptible to viral infection and might be at increased risk for COVID-19. They have a poorer prognosis than healthy individuals because of their systematic immunosuppressive status caused by the tumor burden and anticancer treatments, such as surgery or chemotherapy [35]. The report indicated that the fatality rate for COVID-19 patients with cancer was 7.6% vs. fatality rate of 3.8% in the entire COVID-19 population [39]. Data from different countries are displayed in Table 2 [5, 6, 8, 9, 15, 17, 18, 20, 23–25, 27, 30–33, 36, 37, 40, 42, 44, 45, 47, 48] Chen et al. found that 2019nCoV is more likely to infect older adult males with chronic comorbidities as a result of the weaker immune functions [7]. Liang et al. concluded that among patients with cancer, age is the only risk factor for the severity of the illness [22]. Dai et al. reported that cancer types, stage and treatments may contribute to the severity of the diseases among patients with cancer. However, when patients with cancer only had early stage malignancy without metastasis, the authors failed to observe any difference between the cancer and non-cancer population in terms of COVID-19 related death rate or severity [12]. Liang et al. showed that, compared to patients without cancer, cancer patients were older, more likely to have a history of smoking, had more polypnea, and more severe baseline computed tomography (CT) manifestation. They however had no significant differences in sex, baseline symptoms, other comorbidities, or baseline severity of x-ray [22].

**Table 2:** Mortality rate for COVID-19 patients with cancer

References	Country	Study Design	Deaths	Total	%
Wang J et al [36]	China	Multicenter, retrospective cohort	50	283	17.7
Zhang J-X et al [47]	China	Single-center retrospective cohort	1	14	7.1
Zhang H-Y et al [44]	China	Multicenter, retrospective cohort	18	67	26.9
Zhang L *et al* [48]	China	Multicenter, retrospective cohort	8	28	28.6
Yang K-Y *et al* [40]	China	Multicenter, retrospective cohort	40	205	19.5
Zhang H-Y et al [45]	China	Multicenter, retrospective cohort	14	37	37.8
Meng Y-F *et al* [24]	China	Single-center retrospective cohort	32	109	29.4
Chen T-L *et al * [9]	China	Single-center retrospective cohort	1	7	14.3
Chen T *et al * [8]	China	Single-center retrospective cohort	5	7	71.4
Guan W-J *et al* [15]	China	Multicenter, retrospective cohort	3	18	16.7
Wang L et al [37]	China	Single-center retrospective cohort	3	15	20.0
Basse C et al [5]	France	Single-center, prospective cohort	26	141	18.4
Nikpouraghdam M *et al* [27]	Iran	Single-center retrospective cohort	1	17	5.9
Trapani D *et al* [33]	Italy	Single-center retrospective cohort	150	909	16.5
Rossi PG *et al* [31]	Italy	Multi-center retrospective cohort	44	301	14.6
Benelli G et al [6]	Italy	Single-center retrospective cohort	9	33	27.3
Yarza R *et al* [42]	Spain	Multi-center retrospective cohort	16	63	25.4
Russell B et al [32]	UK	Single-center retrospective cohort	34	156	21.8
Joharatnam-Hogan N et al [17]	UK	Multicenter, retrospective cohort	6	26	23.1
Lee L-Y *et al* [20]	UK	Multicenter, prospective cohort	226	800	28.3
Miyashita H *et al* [25]	US	Multicenter, retrospective cohort	37	334	11.1
Mehta V *et al* [23]	US	Single-center retrospective cohort	61	218	28.0
Kuderer NM *et al *[18]	US	Multicenter, retrospective cohort	121	928	13.0
Robilotti EV et al [30]	US	Single-center retrospective cohort	39	423	9.2

During the COVID-19 pandemic, the utmost risk for patients with cancer is limited access to required health care and inability to receive necessary medical services in a timely fashion, especially in high-risk epidemic areas like Wuhan, where there is a high demand on medical faculty and health care facilities [35]. In the survey, responders reported a 60% decline in clinical volume which implies that there were gynecologic cancer patients who were unable to receive prompt medical attention until COVID-19 fades. A majority of responders (52.8%) believed that ovarian cancer will be the gynecologic cancer most affected by COVID-19. This was followed by uterine cancer (30.0%), and cervical cancer (14.8%) [26]. Ovarian debulking surgery was ranked as the treatment of most concern during the pandemic. This combined with the frequent need for chemotherapy could likely explain why ovarian cancer was rated as the most impacted gynecologic cancer by COVID-19, and thus a particular challenge.

On January 23, 2020, Wuhan began to be locked down, and most departments of gynecology were closed and surgeries and chemotherapy for ovarian cancer were suspended. By the middle of February, some patients have not been treated with chemotherapy for more than 6 weeks. The long interval led to tumor progression. In order to facilitate timely treatment, Wuhan opened 9 designated large-scale general hospitals for covid-19 free cases on February 16, and that figure rose to 40 on March 18th. Divisions of gynecology joined efforts with infection control divisions to transform the ward, strengthen the protection, and provided patients with necessary education about disease prevention and control. In China, the governmental guideline demands that patients with confirmed COVID-19 positive be transferred to hospitals designated by the local governments for COVID-19 treatments.

To date, the pandemic situation in China has improved significantly, with both the number of new cases and fatality markedly reduced. The spread of COVID-19 in Wuhan was effectively controlled and patient care, including cancer treatment, gradually returned to normal. To ensure effective treatment of patients with cancer while avoiding cross infection of COVID-19, greater attention was paid by clinicians to the complex condition of different COVID-19 epidemic phases. Therefore, it is of significance to summarize the relevant experience in the prevention, control of COVID-19 infection and treatment of cancer patients in Wuhan during the epidemic.

### Managements were adjusted in light of the local epidemic situation

During the epidemic, it is important that cancer treatment be adjusted according to the local epidemic status so as to reduce the risk of tumor progression as well as COVID 19 infection. In regard to patients and hospitals in areas with serious or high-risk epidemic situation, such as Wuhan before COVID-19 was under control, strict precautions against the infection and spread of the epidemic was the first priority, and then followed by cancer treatment. Doctors and patients used online medical service to assess whether chemotherapy or surgery should be postponed. Medical personnel paid greater attention to patients after radiotherapy and chemotherapy and discriminated side effects associated with tumor treatment from possible symptoms of covid-19 infection, including fever, vomiting and diarrhea.

In the early stage of the epidemic, patients and health care providers were at higher risk for nosocomial infection, and it was important for hospitals to develop an admission screening process. First, temperature tests were performed at the entrances of the hospital, the outpatient clinic and the wards, and contacts and travel histories of all individuals in the epidemic area were recorded. Second, for patients preparing for hospital admission, compulsory routine blood tests, COVID-19 virus nucleic acid tests and IgM/G, high-resolution CT scans of the lungs were performed. Third, the confirmed cases were transferred to a designated hospital. Patients excluded from having COVID-19 received cancer treatment. Before admission, patients awaiting surgery were contacted by telephone and checked for symptoms related to covid-19 and referred for swabs 48 h before surgery (results available after 24 h). In the case of a negative swab, the interview was repeated before surgery by medical personnel on the day of admission. Treatment was either postponed or cancelled only in cases of suspected covid-19 infection. In addition, at least 7 days prior to cancer treatment, cancer patients stayed in the observation ward and in isolation from other patients. To date, this strategy has permitted clinicians to adhere to surgical protocols for ovarian cancer [43, 48].

As to areas with relatively mild or low risk of epidemic, it was recommended to guarantee timely cancer therapy for tumor patients while preventing and controlling the epidemic. Medical institutions in non-epidemic areas in compliance with the requirements of prevention and control could appropriately open their gynecologic cancer clinics. With the gradual improvement of domestic epidemic situations, patients in non-epidemic areas followed up the original treatment plan, and were encouraged in the use of online treatment. When these patients were assessed for hospitalization, they were screened for reduction of nosocomial infection risk. For example, in case 3, the first symptom of covid-19 was in the digestive tract, and then followed by systems in the respiratory tract. Although there were no detectable abnormalities in lung CT examination, the new coronavirus nucleic acid RNA was detected and COVID-19 infection was diagnosed [46]. Therefore, it is necessary to be vigilant for patients with atypical coivd-19.

With the joint efforts of medical staff around the country, the epidemic has been well controlled in China. Recently, there is an increasing concern about asymptomatic COVID-19 carriers, particularly in one with ovarian cancer, as reported by Chen [10]. A 61-year-old female patient diagnosed as advanced ovarian cancer received a cytoreductive surgery plus intraperitoneal chemotherapy and subsequent first-line chemotherapy for 5 cycles. After the lockdown of Wuhan on January 23, 2020, the patient experienced a short therapy delay. On Feb 10, she complained of a slight dry cough without any other symptoms. Thereafter, she resumed hospitalization for the second-line chemotherapy owing to a poor control of CA-125. On March 21, screening of COVID-19 showed positive serum IgG antibodies to SARS-CoV-2, but negative on serum IgM antibodies, pulmonary CT and throat swab. It has been reported that, with the rapid spread of the disease, infections were diagnosed in individuals who had no direct contact with others with confirmed COVID-19 positive. Yang et al. demonstrated that only one of the three affected gynecological cancer patients was from the metropolitan Wuhan, and the others were from nearby [41]. In an epidemic area of COVID-19, more rigorous surveillance for SARS-CoV-2 should be performed before surgery. After surgery, if a patient develops symptoms of fever or is accompanied by cough, fatigue, sore throat, and/or diarrhea, etc., COVID-19 testing should then be implemented.

With the changing epidemic situation, hospitals in non-epidemic areas should pay special attention to the risk of imported covid-19 [12, 48]. For patients from high incidence areas, isolation observation was carried out in strict accordance with the requirements of the government departments. For suspected or confirmed cases of covid-19, we first treated covid-19 prior to starting cancer treatment. During the COVID-19 outbreak, many healthcare centres were confronted with high demand on pharmaceutical, equipment, and medical supplies, resulting in critical shortages. During this crisis, it was necessary to apply medical ethics in the clinical practice, aiming to treat and cure as many patients as possible with the best use of the available resources [34].

### Ovarian cancer management strategy was formulated according to pathological type, tumor stage and current treatment phase

**Fig. 1:**
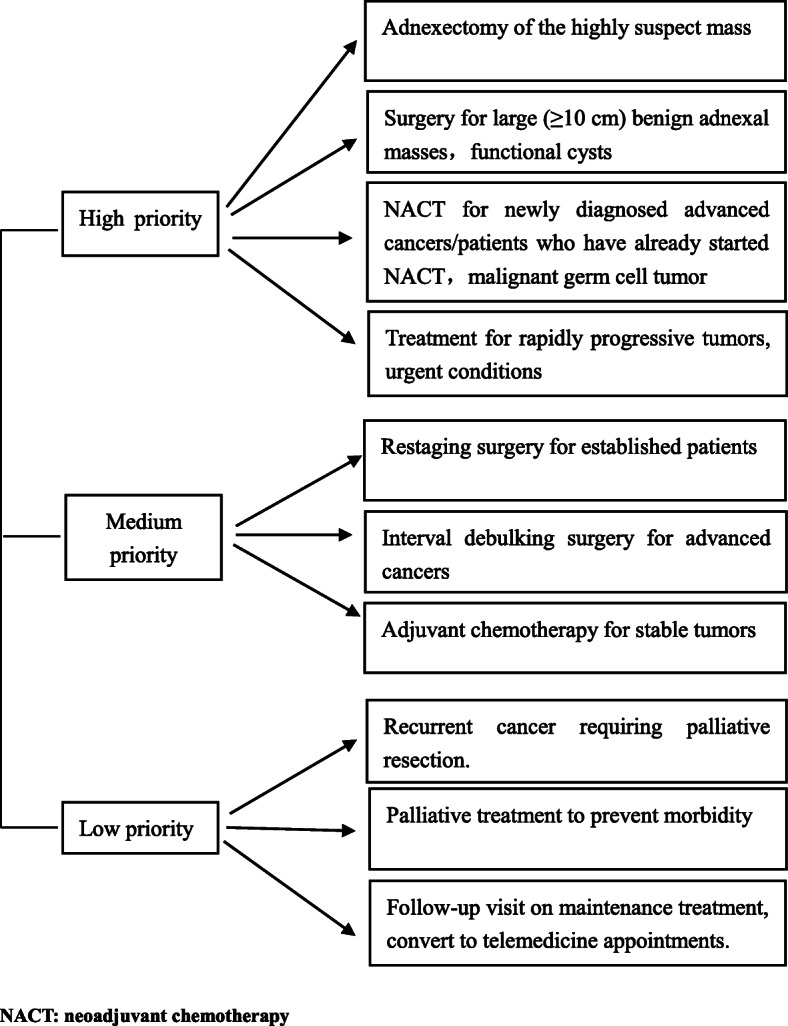
Framework for prioritizing clinical management of ovarian cancer patients during COVID-19 pandemic

Although the treatment plan could be adjusted in the face of the epidemic, the therapeutic principles should remain unaltered. Ovarian cancer continues to be the leading cause of death in gynaecological malignancy worldwide. While covid-19 would have a significant impact on the diagnosis and treatment of ovarian cancer, the management strategies of these cancer patients were still determined by the tumor pathological type and the current treatment phase (Fig. 1). As in case 2 described by Zhang [46], surgery was the first line of treatment for ovarian cancer. However, blood supply and ICU (Intensive Care Unit) support could not be guaranteed during the epidemic period, necessitating neoadjuvant chemotherapy be first introduced to restrain tumor growth until the epidemic eases.

For presumed early stage ovarian cancers according to adnexectomy, restaging surgery could be deferred for 1 to 2 months if access to anesthesia-resuscitation was insufficient. Furthermore, in case of lack of anesthesia-resuscitation, a 2-step strategy was recommended for images suggestive of ovarian cancer on a solitary ovarian mass: adnexectomy of the suspect mass and decision to perform complete staging surgery on ultimate histologic analysis. In highly suspicious early-stage ovarian cancer, deferral surgery was not needed. It was recommended that the patient be referred to an oncologic COVID-19-free center after a complete evaluation that includes vaginal examination, MRI, transvaginal ultrasound, and serum markers (CA125). Intraoperative frozen section analysis was imperative to confirm the diagnosis. Surgical treatment of early-stage ovarian cancer was the standard of care by international guidelines [3, 11]. Young (< 30 years) asymptomatic patients with large (≥10 cm) benign adnexal masses must be submitted to surgery. Medical therapy is recommended in patients with apparently functional cysts unless it is contraindicated.

For advanced cancers which may require the use of postoperative recovery for cytoreduction surgery, neoadjuvant chemotherapy (NACT) should be the preferred choice even if primary cytoreduction surgery could be envisaged. If access to the operating room was restricted due to the crisis, patients scheduled to receive interval surgery after 3 or 4 cycles of chemotherapy could continue their chemotherapy and have surgery after 6 cycles of chemotherapy. The patient should then undergo at least two new cycles of chemotherapy after their closing surgery. Similar proposals were suggested by a multinational group of practitioners that put emphasis on NACT for advanced ovarian cancer even for tumors estimated to be resectable in order to decrease high risk surgeries and long ICU stays [1, 14, 29]. Another Chinese study identified the lack of strict guidelines for the management of gynecological cancer in an endemic region [38].

For chemotherapy-based patients, such as those with malignant germ cell tumor, one should give priority to the needs of these patients for chemotherapy; otherwise the tumor would progress more rapidly [43]. Physicians should determine whether chemotherapy could be temporarily delayed or changed, such as changing weekly treatment to a three-week duration and suspending radiotherapy sensitization chemotherapy to reduce the number of hospital visits. We also suggested that patients from other locations be treated in local hospitals, so as to divert patients and to reduce the aggregation of personnel in large hospitals.

With regard to newly diagnosed patients, tumor progression is more likely to be life-threatening. With the premise of guaranteeing the requirements of epidemic prevention and control, appropriate priority was given to ovarian tumor patients with relatively rapid tumor progression, and attention was paid to avoid tumor rupture, bleeding and other emergencies. Active cancer treatment during the epidemic period can prevent the rapid progress and fatality of the tumor. Although covid-19 infection might have occurred, it also gained time for the following cancer treatment. For example, it was completed as in case 3 reported by Yang [41]. However, patients in consolidation chemotherapy stage, or patients with tumor recurrence, whose short-term threat to life was relatively small or delayed treatment had little impact on prognosis, one could postpone treatment until conditions permit.

If the chemotherapy or surgery had to be performed after evaluation, the patients were admitted to the hospital for treatment as soon as possible under the condition of adequate protection. Some cancer patients could also have acquired COVID-19 infection on receiving cancer treatment during hospitalization. However, delaying cancer therapy could not be recommended as a reasonable choice to reduce the infection risk in the ongoing pandemic. Stronger personal protection, including protection for their families, were made for cancer patients [22].

### Specific considerations and the worldwide imposed treatment modifications for ovarian cancer during COVID-19 era

To date, the world has been fighting against COVID-19 since December 2019. The oncology community is being challenged, requiring a timely response to a fast-changing situation. Noteworthy modifications such as postponing elective operations, increasing rates of NACT in advanced illness, reducing/suspending surgery at recurrence and transforming to online consultation were recommended. The general guiding principle of these recommendations is the adoption of a “do no harm” method [1, 3, 11]. Nogami Y et al. demonstrated their experience in Tokyo. The surgery of an asymptomatic RT-PCR-positive patient was delayed by 4 weeks until the confirmation of negative results. The other two patients were RT-PCR-negative, but abnormal CT findings indicated the possibility of COVID-19, which delayed treatment. The patient who received NACT for ovarian cancer had clinically apparent deterioration because of the treatment delay [28]. Kobayashi Y et al. present two ovarian cancer cases whose treatment plan were affected by the local circumstances of COVID-19 in Daegu [19].

Frey MK and her colleagues revealed that types of surgery performed were different (*p* = 0.034), with fewer cytoreductive surgeries for ovarian cancer and laparoscopic procedures (*p* = 0.002) in 2020 [14]. Altın D et al. reported that most Turkish gynecologic oncologists modified their treatment of gynecologic cancers due to the COVID-19 pandemic. In Turkey,67.1% of surgeons operated early stage ovarian cancer patients, 50% implemented NACT to all advanced stage ovarian cancers and 50% administered more cycles of NACT prior to interval debulking surgery [2].

It has been proposed that gynecologic cancer surgery should continue during the COVID-19 pandemic while complying with the established infection control measures. Postponement or non-surgical management should only be considered in patients with identified infection [4, 13]**.** Leung et al. held that maintaining surgical volume with detailed planning by a cohesive multidisciplinary team is practicable during the COVID-19 pandemic but this was associated with an increase in postoperative complications owing to a number of reasons [21].

The European Network of Gynaecological Cancer Advocacy Groups (ENGAGe) conducted a survey in 16 European countries and found that gynaecological cancer patients expressed significant anxiety about progression of their disease due to modifications of care related to the COVID-19 pandemic and wished to pursue their treatment as planned despite the associated risks [16].

## Conclusions

The current COVID-19 pandemic has imposed excessive burdens to the healthcare system. Priority should always be given to the life-threatening circumstance. Treatment cancellation and delaying surgeries are challenging decisions. When confronted with limited resources during the COVID-19 pandemic, a multidisciplinary cancer treatment team (which may include medical ethicists and palliative care specialists) must decide which patients will receive complicated or critical care based on the expected clinical outcomes [27]. Relatively younger age, presumed immune-compromised and delay in cancer remedy were associated with significantly higher levels of cancer worry, anxiety and depression. Healthcare experts should consider or incorporate patients perspectives when making decisions that impact patients care during the pandemic crisis.

Furthermore, ensuring the continuum of care for ovarian cancer patients is crucial and is considered a major priority during the epidemic. Therapeutic regime of ovarian cancers should be based on patients individual characteristics, taking into consideration local COVID-19 infection situation and accessibility of medical facilities. Prognosis is also a crucial consideration if delay is contemplated. Medical institutions are recommended to make full use of the internet medical service platform to establish rapid online consultation, health assessment, and education. Experts could also be organized to carry out online health consultation, disease prevention and control lectures, so that the patients are aware of their own health status and receive reasonable and orderly treatment under professional guidance. However, in case of cancer progression or worsening of the disease, patients should visit an oncologist rather than seeking cure through online consultation. Evidence in regard to these recommendations is limited because of the novel and unknown nature of the COVID-19 pandemic. Furthermore, data in relation to ethical debates about delayed therapy and therapy deviating from current guidelines are also in shortage. There is an urgent need for well-designed trials to identify the clinical outcomes of continuing or withholding cancer therapy and the proper prevention, management, and treatment of COVID-19 in the oncology settings.

So far, China has continued to implement actions to control any resurgence of new cases of COVID-19. Despite the challenges encountered during the COVID-19 pandemic, we believe that appropriate shielding and screening protocol in place for our patients accompanied by a sturdy mitigation plan to protect surgical and medical faculty would ensure the delivery of safe ovarian cancer treatment service.

## Data Availability

Not applicable.
